# MoSART: Mobile Spatial Augmented Reality for 3D Interaction With Tangible Objects

**DOI:** 10.3389/frobt.2018.00093

**Published:** 2018-08-20

**Authors:** Guillaume Cortes, Eric Marchand, Guillaume Brincin, Anatole Lécuyer

**Affiliations:** ^1^REALYZ, Louverné, France; ^2^University of Rennes 1 (Univ Rennes), Inria, CNRS, IRISA, Rennes, France

**Keywords:** 3D Interaction, spatial augmented reality, optical tracking, tangible objects, head-mounted display

## Abstract

In this paper we introduce MoSART, a novel approach for Mobile Spatial Augmented Reality on Tangible objects. MoSART is dedicated to mobile interaction with tangible objects in single or collaborative situations. It is based on a novel “all-in-one” Head-Mounted Display (AMD) including a projector (for the SAR display) and cameras (for the scene registration). Equipped with the HMD the user is able to move freely around tangible objects and manipulate them at will. The system tracks the position and orientation of the tangible 3D objects and projects virtual content over them. The tracking is a feature-based stereo optical tracking providing high accuracy and low latency. A projection mapping technique is used for the projection on the tangible objects which can have a complex 3D geometry. Several interaction tools have also been designed to interact with the tangible and augmented content, such as a control panel and a pointer metaphor, which can benefit as well from the MoSART projection mapping and tracking features. The possibilities offered by our novel approach are illustrated in several use cases, in single or collaborative situations, such as for virtual prototyping, training or medical visualization.

## 1. Introduction

The use of Augmented Reality technologies has been proposed in many application fields so far, such as for industrial, medical or robotic applications (Van Krevelen and Poelman, 2010). Augmented Reality (AR) consists in superimposing virtual content over real objects. Three main approaches have already been proposed in AR to display virtual content over the real environment.

The most affordable and mainstream approach is probably the Video See-Through AR (VST-AR) (Mohring et al., 2004). This first technique consists in adding virtual information over a regular video stream. In general, VST-AR relies on smartphone technologies, making it a simple and accessible way to provide AR to the general audience. Thus video see-through systems are commonly used in many applications (Schmalstieg and Hollerer, 2016). Video see-through systems are either hand-held or head-mounted. Hand-held devices are generally not well-adapted for direct interaction with the hands, whereas Head-Mounted Displays (HMD) are known to be sensitive to latency.

A second AR technique is the Optical See-Through approach (Olwal et al., 2005) that has been recently democratized with systems such as the Microsoft Hololens^1^. Optical see-though AR (OST-AR) consists in displaying virtual content on near-eye semi-transparent screens, so that the real world can be directly looked at. Such systems often have low latency, accurate positioning and are well-designed for interactive environments by freeing the user's hands. However OST-AR approaches often fail at providing a large Field-Of-View (FOV), and are not well-designed to comply with multi-user applications.

The third technique is called Spatial Augmented Reality (SAR) or Projection-based AR (Bimber and Raskar, 2005). SAR systems are based on a direct projection over real physical surfaces through projection mapping. This technique enables a larger field-of-view with a reduced latency, and shared experiences with other people. But SAR systems are mostly static (due to the use of a projector) which often restricts mobility. Hand-held devices exist but, similarly to VST-AR, they are intrinsically limited in terms of direct interaction.

**Figure 1:**
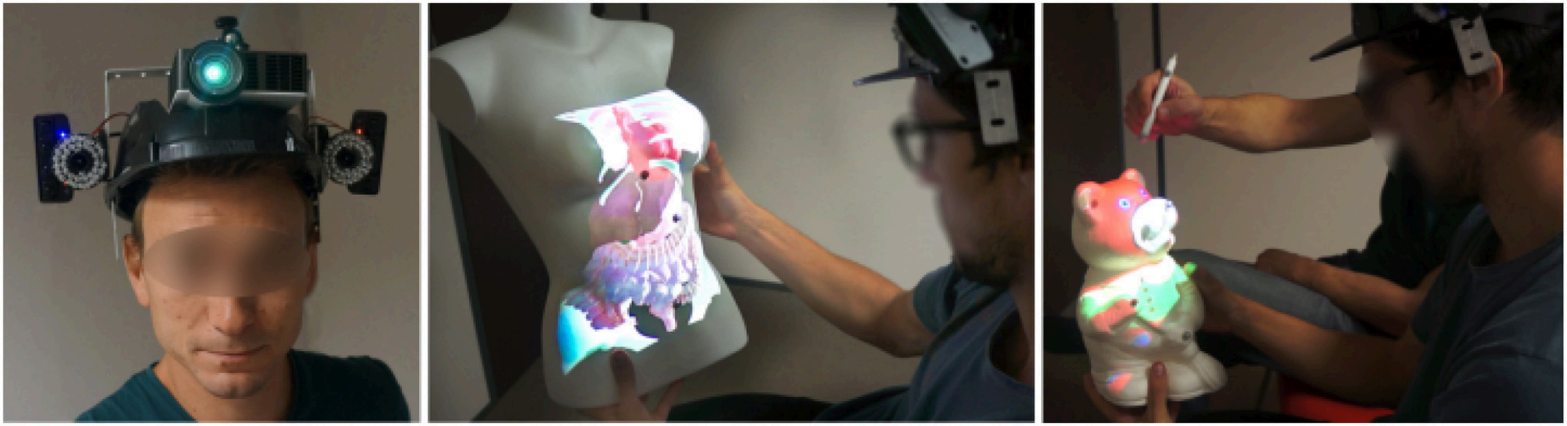
Our MoSART approach enables Mobile Spatial Augmented Reality on Tangible objects. MoSART relies on an “all-in-one” Head-Mounted-Display **(Left)** which embeds a pico-projector for projection mapping and two cameras for feature-based stereo optical tracking of 3D tangible objects. The user can freely walk around and manipulate tangible objects superimposed with the projected images, such as for medical visualization purposes **(Center)**. Tangible tools can also be used to interact with the virtual content such as for annotating or painting the objects in single or collaborative scenarios **(Right)** [This image is published with the written and informed consent of the depicted individual(s)].

In this paper, we promote an alternative approach for head-mounted SAR which enables mobile, direct and 3D interaction with real tangible objects, in single or collaborative scenarios. Our novel approach, called MoSART (for Mobile Spatial Augmented Reality on Tangible objects) is based on an “all-in-one” headset gathering all the necessary AR equipment (projection and tracking systems) together with a set of tangible objects and interaction tools (Figure 1). The tangible objects and tools are tracked thanks to an embedded feature-based optical tracking providing 6-DOF (degrees of freedom) positioning data with low latency and high accuracy. The user can walk around, grasp and manipulate the tangible objects and tools augmented thanks to projection mapping techniques. Collaborative experiences can be shared with other users thanks to direct projection/interaction. In a nutshell, our approach is the first one which enables direct 3D interaction on tangible objects, mobility, multi-user experiences, in addition to a wider field-of-view and low latency in AR.

To summarize, the main contributions of this paper are:

- MoSART, a novel approach for SAR that can simultaneously enable: mobile SAR on movable objects, 3D interactions with tangible objects, single and/or collaborative scenarios and experience sharing in AR applications.
- An operational prototype of the MoSART concept based on: (1) a novel all-in-one headset gathering head-mounted projection and optical tracking, and (2) a set of tangible objects and interaction tools.
- Several use cases that illustrate the potential of MoSART in different application contexts such as virtual prototyping and medical visualization.

In the remainder of this paper we first present an overview of previous work on SAR. Second, we describe the MoSART concept for mobile spatial augmented reality on tangible objects. Third, we present a proof-of-concept with the design of a MoSART prototype, and we assess its main characteristics and performances. Fourth, we describe two use cases of MoSART for virtual prototyping and medical visualization purposes. The paper ends with a discussion and a general conclusion.

## 2. Related work

Spatial Augmented Reality systems are generally used to project textures and augment stationary objects (Aliaga et al., 2012; Siegl et al., 2015). Projecting on stationary objects with a stationary system gives good performances once everything is correctly calibrated. Nevertheless the use cases of such systems can be limited and few mobility or direct interactions can be considered. Thus more dynamic systems were designed to augment movable (Zhou et al., 2016) or deformable (Punpongsanon et al., 2015) objects and to propose interaction techniques. Work from Hochreiter et al. (2016) proposes multi-touch detection for interacting on augmented stationary objects directly with the fingers. Benko et al. (2012) proposed the Miragetable: a dynamic spatial AR system with projection on a curved surface. These systems widen the possibilities of interaction since the real environment and the user motions are taken into account. However, since the projection is made on a stationary screen (or object) the usable workspace is rather limited. To overcome such limitation several spatial AR system were designed to project on movable 3D objects. The Lumipen, designed by Okumura et al. (2012), provides projection mapping for high-speed or high-frequency objects thanks to an high-speed vision sensor and a projector with an high-speed optical gaze controller. The Lumipen works well on simple 3D objects such as spheres and balls due to the insignificance of their rotation. In more recent work, Sueishi et al. (2015) proposed an improvement of the Lumipen. Nevertheless their system is far too cumbersome and is still used on simple geometries. Such limitations do not provide an ideal environment for tangible interaction. Zhou et al. (2016) proposed the Pmomo: a projection mapping system on movable objects. The Pmomo handles more complex geometries with acceptable tracking performances. Even though the system is lighter than the previous approaches it is still stationary and is not designed to be portable or embedded. Moreover the current version of the system does not enable tracking several objects which can be inconvenient in many interaction scenarios. To compensate the limitations of a stationary projector work from Benko et al. (2015) proposes to combine it with OST-AR and provide more freedom to the user with a larger field of view induced by the projection. Nevertheless this approach is interesting whenever the user is in the workspace of the projector. Indeed outside of this workspace the field of view becomes limited again by the OST-AR system.

A first approach to overcome stationary systems is to design hand-held devices. With hand-held devices the projector needs to have knowledge of the geometry of the scene since it needs to be aware of its localization at each instant. Work from Raskar et al. (2006) introduces the iLamps, geometrically aware projector. The approach is illustrated with a hand-held projector and single-user applications. Hand-held projectors have been studied in several posterior works. In 2007, Cao et al. (2007) introduced multi-user interactions with two projectors that are tracked with feature-based tracking. The users can interact by moving the projectors in the workspace with a visual feedback projected on a flat wall. Still, the interactions are limited to planar objects and no 3D is considered. Ni et al. (2011) introduced hand-held projection in medical applications to improve doctor-patient communications. With such system the doctor is able to project anatomical information directly over the patient body. Nevertheless the authors pointed out that the proposed system was more usable when projecting on a wall. More recent work has been proposed based on same approach with the SideBySide system (Willis et al., 2011). The SideBySide system tracks several projector that project fiducial markers on a wall but the system is not adapted to tracking 3D tangible objects. Even though hand-held SAR devices provide more mobility than stationary SAR systems they are not adapted to direct interactions since the user's hands are not free. A french company, Diota^3^, proposes a SAR device that is able to move without being held in the hand. This solution is based on robotic arms that move the projectors around the objects. Nevertheless such solution is not designed to be portable or to be used in small indoor environments.

Since holding the projector in the hand is not always satisfying, work has been done to project from the head of the shouder. Nevertheless mounting a projector on the head (or shoulder) can be more complicated due to the weight it induces. One of the first work going in that direction has been carried out by Karitsuka and Sato (2003). They propose a shoulder-mounted projection system to augment a planar target. Then the user is able to interact with the augmented planar target by using his/her fingers. Bolas and Krum (2010) introduced head-mounted projection on reflective surfaces. Nevertheless they do not introduce interaction techniques for augmented reality and they only project informative content that cannot be modified. CastAR^3^, a start-up company, implemented an industrial product based on head-mounted SAR. Their system projects 3D images over reflective surfaces that can have different predefined simple shapes and enables the user to interact with the environment. The prototypes proposed by CastAR get close to a virtual reality projective holobench system and they do not propose any augmentation of tangible 3D objects. Unfortunately CastAR closed their doors in 2017 due to a lack of interest for this technology in the industry they were targeting. Work from Akşit et al. (2014) also proposes an approach to project on planar surfaces from an head-worn mixed reality system based on a laser pico-projector and a smartphone. But unlike CastAR the authors chose to focus motion capture application. Thus the system is prototyped to work in a larger and non-friendly infra-red environment. However the projection over 3D tangibles objects is still not considered and no tracking system is required other than the smartphone sensors. More recent work from Harrison et al. (2011) introduces a shoulder-mounted system implementing direct hand interaction techniques. Indeed mounting the projector on the shoulder also leaves the hands free to interact. The interaction they proposed is a tactile one on simple surfaces and on body parts. The projection over those surfaces is still planar and the geometry of tangible objects is not taken into account.

## 3. MoSART: mobile spatial augmented reality on tangible objects

We introduce MoSART, a novel approach for mobile spatial augmented reality on tangible objects. MoSART enables mobile interactions with tangible objects by means of head-mounted projection and tracking. MoSART allows to straightforwardly and directly manipulate 3D tangible objects, and then interact with them using dedicated interaction tools. It also allows sharing the experience with other users in collaborative scenarios.

**Figure 2:**
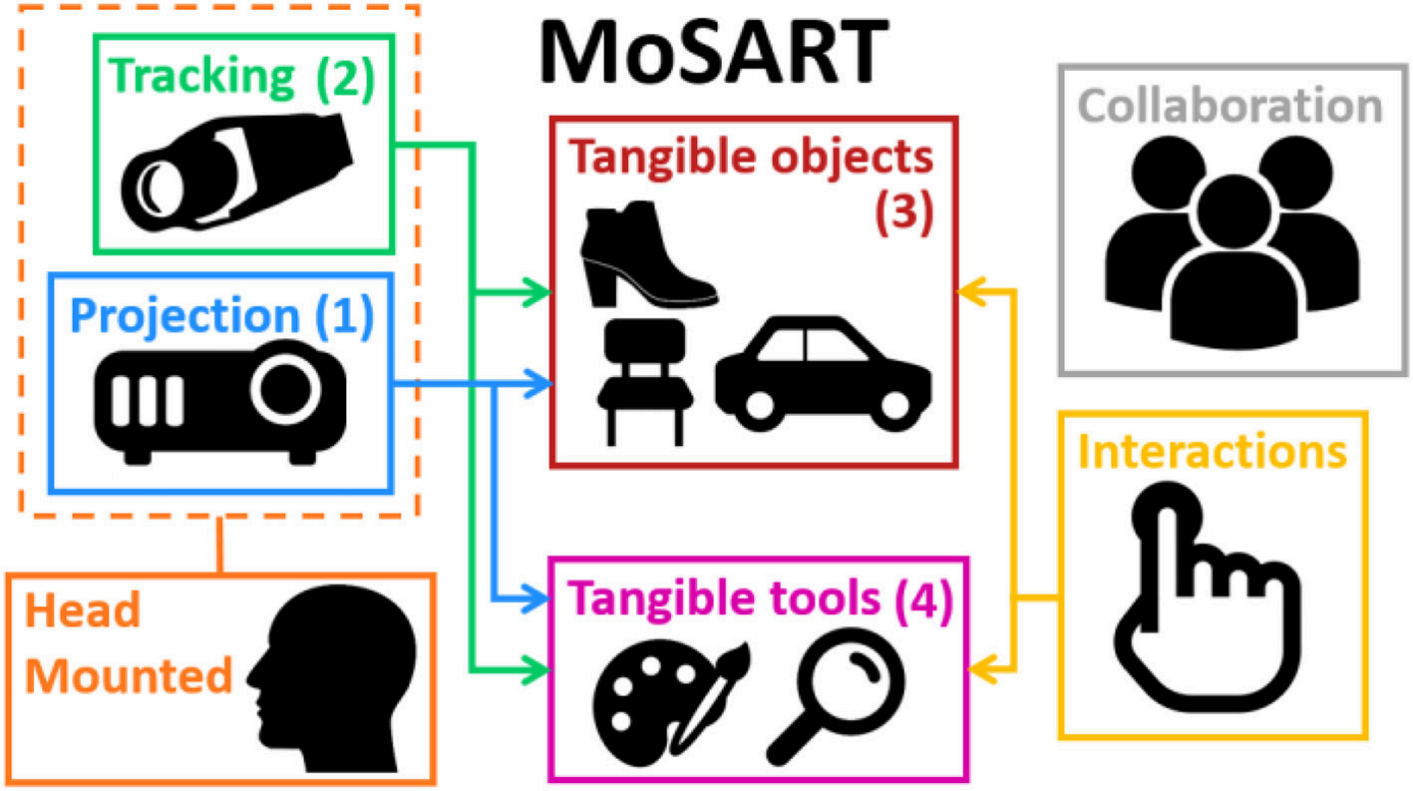
Main components of the MoSART approach. MoSART involves head-mounted projection (1) and tracking (2). Direct 3D interactions are made possible with the tangible objects (3) and tools (4). Collaboration and multi-user scenarios can be addressed with or without additional headset(s).

The main components of a MoSART system are thus: (1) a head-mounted projection, (2) a head-mounted tracking, (3) tangible object(s), (4) several interaction tools. These main components are illustrated in Figure 2 and explained hereafter.

1. **Projection:** Head-mounted projection is used by MoSART to display the virtual content in the field-of-view and workspace of the user in a direct and unobtrusive way allowing to augment the objects located in the user's field of view. This also implies that projection mapping techniques are required to match the 3D surface of the tangible object with the virtual content.
2. **Tracking:** Head-mounted tracking is used to follow the tangible objects and enable their manipulation in the workspace/FOV of MoSART. It enables the user to walk and move around the objects, manipulate (rotate/translate) them at will. This naturally implies that the SAR projector must be intrinsically tracked by the system.
3. **Tangible objects:** The use of 3D tangible objects is at the core of the MoSART approach. Thus the approach requires having both a physical model and a 3D virtual model of the object the user is interacting with.
4. **Interaction tools:** Tangible tools can also be incorporated straightforwardly within MoSART. Such tangible tools can benefit from the projection and tracking features of the system. This means that the tool surface can be used to project virtual content, and that the tools need to remain inside the projection/tracking volume. This also implies that dedicated 3D interaction techniques and metaphors need to be designed for every tool.

- **Head-Mounted:** To free the hands and provide an entire mobility to the user, all the projection and tracking features are mounted on the head.
- **Direct interaction:** Direct 3D interaction is a main advantage of MoSART thanks to the use of tangible objects. With MoSART the user can grasp tangible objects, and then manipulate (rotate/translate) them at will, within the field of view of the projector.
- **Collaboration:** Collaboration and multi-user interactions are a main advantage of MoSART. Two complementary collaborative modes are made possible. First, if there is only one user equipped with a MoSART headset (single-device configuration), MoSART allows other user(s) to share the direct projection controlled by the main user. The other users can also manipulate the tangible object(s) and/or some interaction tool. Second, if other headsets are available (multiple-devices configuration), the different projectors can be used to increase the projection area having for instance one user projecting on one side of the tangible object, and another user projecting on another side.

A prototype of the MoSART concept is introduced in the following section, and implementation details are provided regarding each MoSART component.

## 4. Proof-of-concept

**Figure 3:**
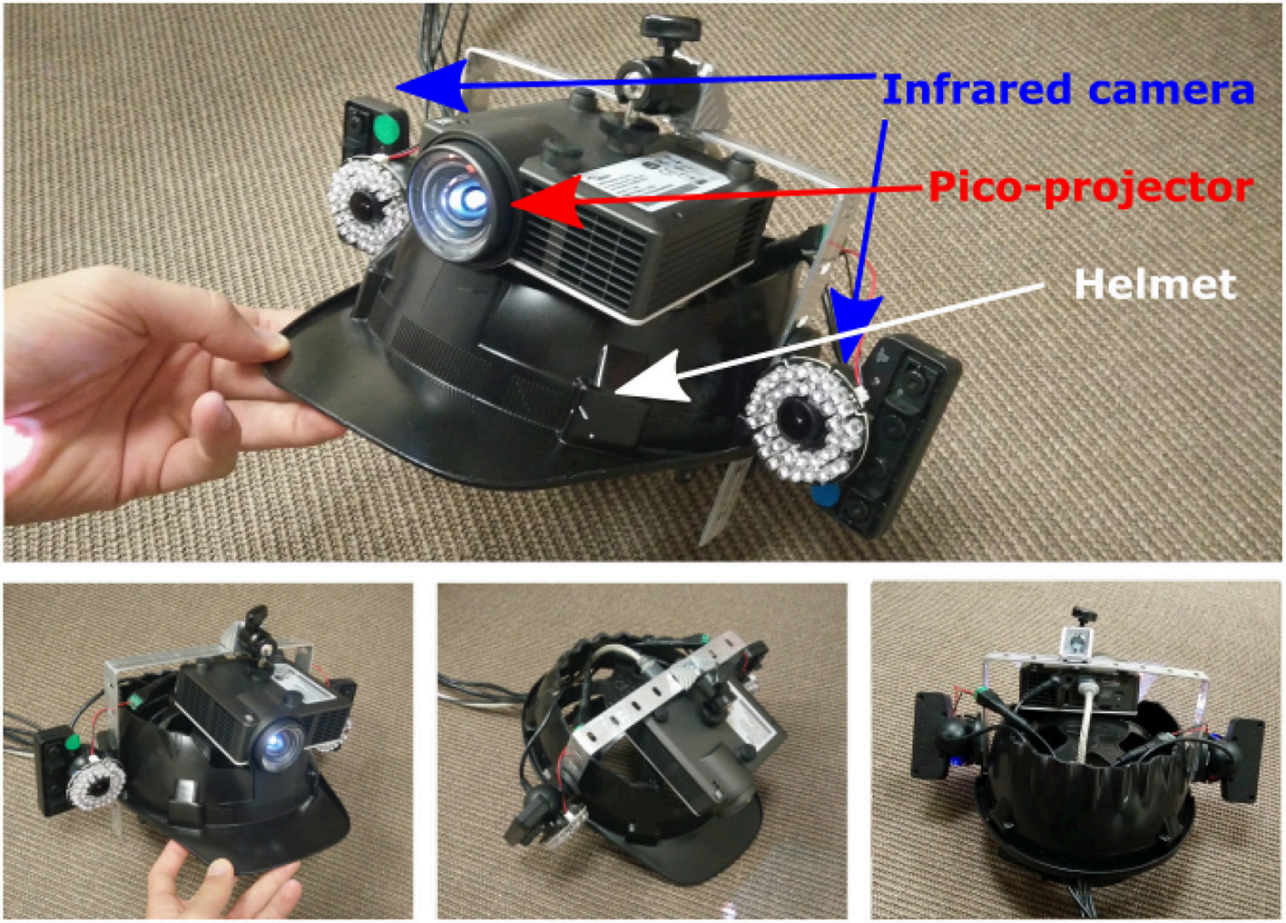
Prototype of MoSART headset. The headset gathers a pico-projector (for projection mapping) and two infrared cameras (for optical tracking).

**Figure 4:**
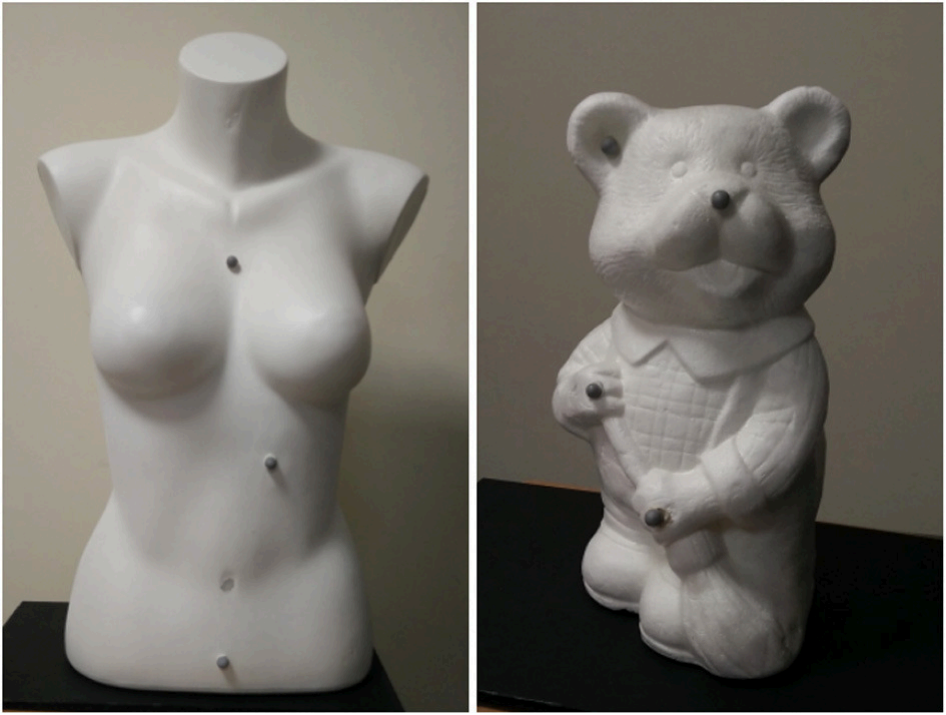
Examples of tangible objects augmented with MoSART. The objects are white or covered with white painting. Reflective markers are positioned over the objects to facilitate their localization.

We have designed a proof-of-concept of the MoSART approach. Our prototype includes a headset (Figure 3) and a specific set of tangible objects (Figure 4) and tangible tools (Figure 8), coming with dedicated 3D interaction techniques.

Our headset gathers one short throw pico-projector (Optoma ML750ST) and two infrared cameras (PSEye). The cameras are rigidly attached on both sides of the projector. The whole projection/tracking system is mounted on the head and it is positioned so that the projection remains centered in the user's vision.

The projector is used to perform projection mapping on the tangible objects that are tracked with the optical tracking system (see Figure 3). The cameras are used to provide 6-DOF tracking data of the tangible objects thanks to feature-based stereo optical tracking algorithms. An off-line initial calibration step is required to estimate the position and orientation of the projector with respect to the cameras. Such a configuration (projector and tracking system attached) allows the system to be moved around the scene. The system does not need to track the projector localization anymore since the relative cameras/projector position is constant.

The tangible objects used are ideally (but not necessary) white or covered with reflective white paint coating, allowing to provide better results in terms of image color and contrast when projecting over the object. Several reflective markers (commonly 4 or 5) are positioned at the surface of every tangible object (see Figure 4), and are used to track and localize it using an optical tracking system.

### 4.1. Optical tracking

The tracking system mounted on the helmet is used to localize the tangible objects and interaction tools. The objective is to compute the position and orientation of the objects according to the projector. The system computes tracking data from the video streams provided by the two infrared cameras and it relies on feature-based stereo optical tracking. Feature-based optical tracking provides generally better performances than model-based tracking techniques in terms of accuracy and jitter. Localizing a rigid structure of markers (constellation) can be done generally faster than localizing a model. Moreover tracking several objects can be straightforwardly achieved by using different constellations for different objects. Also, using markers makes the tracking independent of the geometry of the objects, only the markers' disposition matters. Nevertheless it requires to add physical markers all over the tangible objects. To be able to localize a constellation it requires to have at least 3 markers (typically 4 or 5) and the distances between them have to be all different (Pintaric and Kaufmann, 2007). Such constellation configuration reduces the ambiguities when computing the 3D registration to recover the pose of the objects (see step 4).

The tracking process is performed with offline and online steps. Optical tracking systems usually require an offline calibration process. Such calibration estimates the relative position ^*c*^${\text{M}}_{c^{\prime }}$ between camera *c* and camera *c*′ (see Figure 7) in order to be able to correlate the visual features in each view and to recover the 3D position of each reflective marker. It also estimates the camera internal parameters and distortion coefficients. Once the tracking system is calibrated four main online steps are performed to provide 6-DOF localization of the object (Pintaric and Kaufmann, 2007):

1. The **features extraction** determines the position of the bright markers in the images acquired by the two cameras.
2. The **features correlation** is performed thanks to the offline calibration: the points from one image are associated with their corresponding points in the other image.
3. The **triangulation** allows to recover 3D points coordinates. The computation of the 3D coordinates is derived from their projections in the two image planes knowing the calibration parameters of the system.
4. The **3D registration** estimates the transformation ^*c*^${\text{M}}_{o}$ that defines the pose (position and orientation) of the object in one of the camera frame. This is achieved by minimizing the error between the 3D reconstructed points ^*c*^${\text{X}}_{i}$ (in the camera frame) and the known corresponding 3D points ^*o*^${\text{X}}_{i}$ (in the object frame) transferred in the camera frame through ^*c*^${\text{M}}_{o}$. By denoting $q={{(}^{c}t_{o},\theta u)}^{⊤}$ a minimal representation of ^*c*^${\text{M}}_{o}$, the minimization problem is reformulated:
  (1) $$\hat{\text{q}}=argmin_{\text{q}}\sum_{i=1}^{N}{{(}^{c}{\text{X}}_{i}{-}^{c}{{\text{M}}_{o}}^{o}{\text{X}}_{i})}^{2}.$$
  The problem is solved by initializing the pose, ^*c*^${\text{M}}_{o}$, with a linear solution, based the one proposed by Arun et al. (1987), and refining it with a non-linear Gauss-Newton estimation.

**Figure 5:**
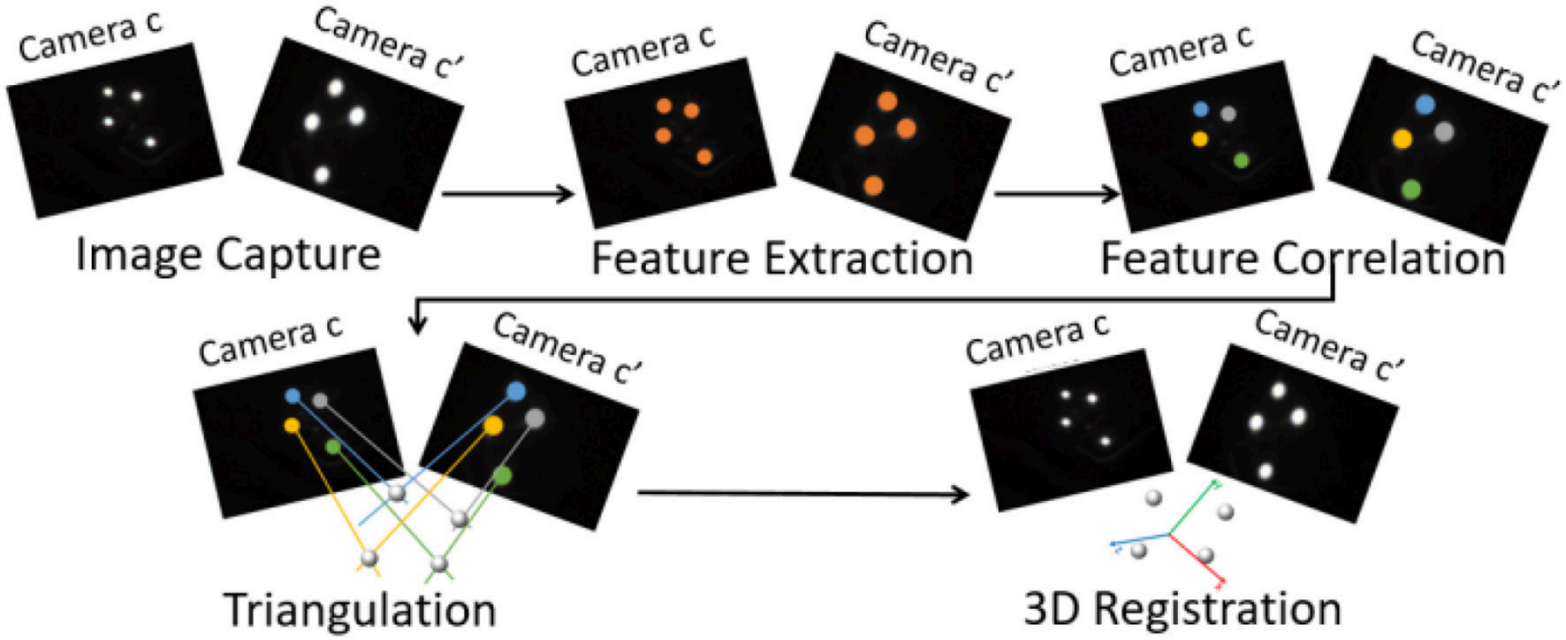
Stereo optical feature-based tracking pipeline. It is performed in four main steps: feature extraction, features correlation, triangulation, and 3D registration.

Figure 5 illustrates the different steps of the online stereo optical tracking pipeline. The tracking of several tangible objects can be performed. Each object pose is sent to update a virtual scene. Thus this virtual scene matches the real environments and the projected image can then be rendered (see section 4.2.3).

The tracking system of the MoSART prototype was built with two Sony PsEye cameras providing 320 × 240 images at 150 Hz. Infrared rings and infrared filters have been added to the cameras to capture the infrared light reflected by the reflective markers and ease the features extraction process.

### 4.2. Projection mapping

**Figure 6:**
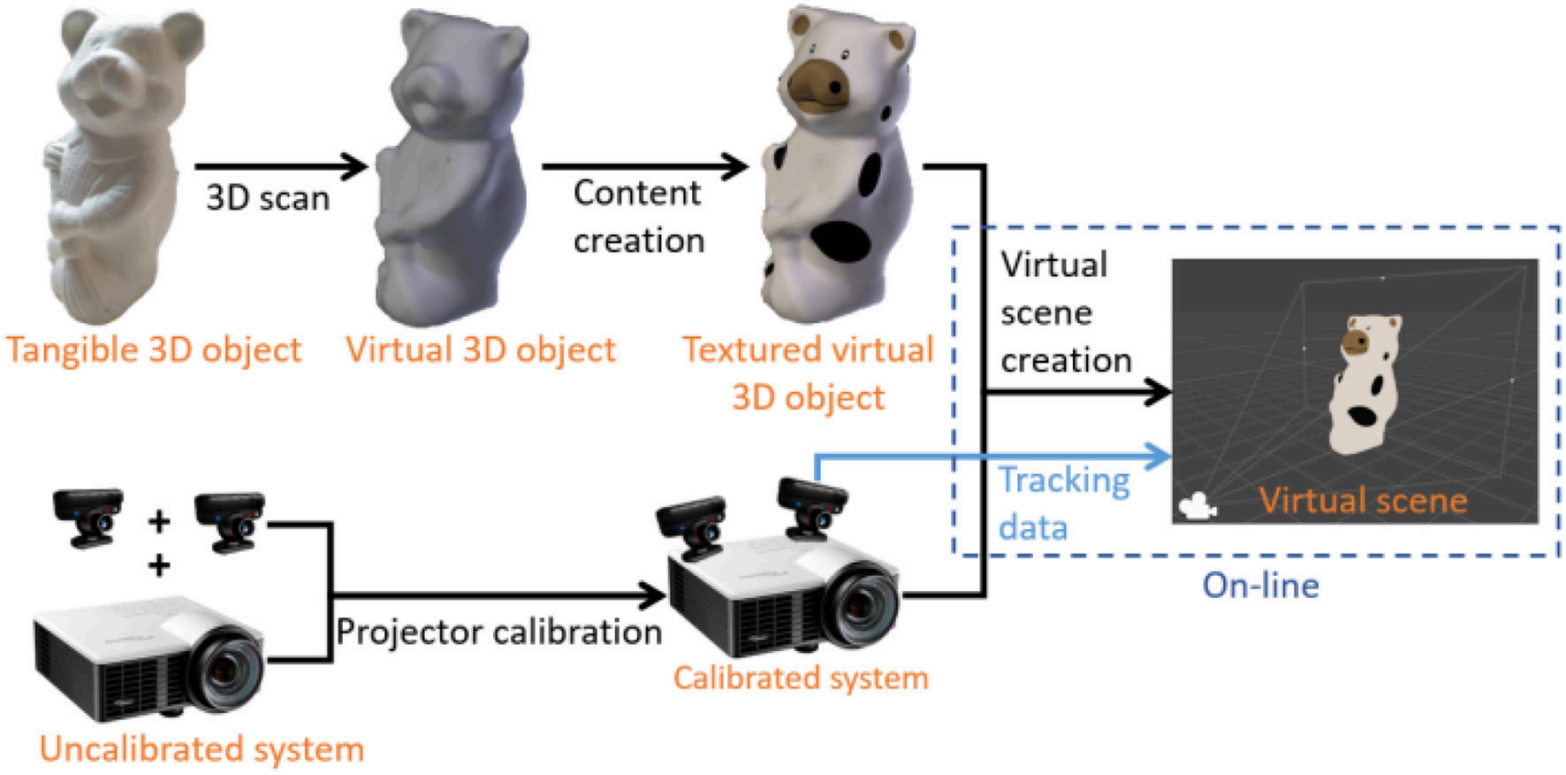
Projection mapping pipeline. The object part **(Top)** is responsible of the virtual object creation including scanning and content creation. The projection part **(Bottom)** is responsible of the projector calibration and projection model estimation. It also involves the tracking systems that localizes everything. A virtual scene is created on-line to match the real environment.

The projection mapping consists in mapping the virtual 2D image of a tangible object to the physical model on the same objects. To achieve this goal the application needs to have full knowledge of the object's shape and of the projection model. The projection model determines how a 3D point is projected into the image frame (3D-2D projection). In this case the projection model of the projector needs to be known to perform an “inverse-projection” (2D-3D projection). An off-line calibration process is used to determine such projection model. Regarding the object's shape, an off-line process is performed to scan and reconstruct the tangible object and incorporate its model in the application. Once both side of the system are known a virtual 3D scene can be generated to exactly fit the real scene. Figure 6 summarizes the projection mapping pipeline. Each step of the pipeline is detailed in the following sections.

#### 4.2.1. Virtual object creation

A first requirement for projection-based augmented reality on a tangible object is to have access to its 3D model. This model is obtained with a 3D scanning technique. A structured light (infrared) depth sensor provides a depth map of the environment where the object is located and by moving the sensor around the object several maps are captured. An Iterative Closest Point (ICP) algorithm is used to match each dense map to the global model. Then the depth maps are fused to build a model of the tangible object (e.g., Newcombe et al., 2011). The 3D model of the object has its own coordinate system that we call virtual object frame ($F_{vo}$). The physical objects has no predefined frame but its frame, $F_{o}$, is defined by the tracking system. For a matching between the virtual scene and the real scene frame $F_{vo}$ and frame $F_{o}$ need to be the same. Thus another ICP algorithm is used to match at least four points of the virtual model to the same four points in the physical model. Once this matching process is carried out the transformation between $F_{vo}$ and $F_{o}$ is known and remains constant.

#### 4.2.2. Projector calibration

The projector calibration is one of the most sensitive steps of projection mapping. Indeed the projection model and the projector pose need to be accurately known to ensure an acceptable mapping. This calibration is carried out once for each system so it needs to be as accurate as possible.

##### 4.2.2.1. Projection model estimation

The projection model of a projector is very similar to a camera model and it consists in a projection matrix and distortion parameters. The projection matrix and the distortions are estimated thanks to a the calibration process (Yang et al., 2016). The projection matrix determine the projection model and how a 3D point is projected into the image. The distortions enable to determine the corrections that need to be applied to the image to perfectly fit the tangible objects of the real scene. Camera-projector calibration algorithms are adapted to this case. The calibration involves a projector and a camera that are stationary relatively to each other. A 9 × 6 black and white chessboard is used for the calibration and several positions of this chessboard are capture by a camera. For each position of the chessboard 4 corners are selected by the user in the projector frame and an homography is computed between the projector frame and the camera frame. This homography is used to find the position of all the remaining corners of the chessboard in the projector frame. With these positions the projection matrix and distortions parameters of the projector can be estimated. The same method is used to calibrate the camera.

##### 4.2.2.2. Projector pose estimation

Once several views of the chessboard have been captured, the pose of the projector in the camera frame, ^*c*^${\text{M}}_{p}$, can be computed from the measurements. Indeed the pose of the camera, ^*o*^${\text{M}}_{c}$, and the pose of the projector, ^*o*^${\text{M}}_{p}$, according to each chessboard position can be estimated with a PnP algorithm on planar objects (Marchand et al., 2016). Then the relative pose between the projector and the camera can be computed with :

(2) Mcp=McoMop.

**Figure 7:**
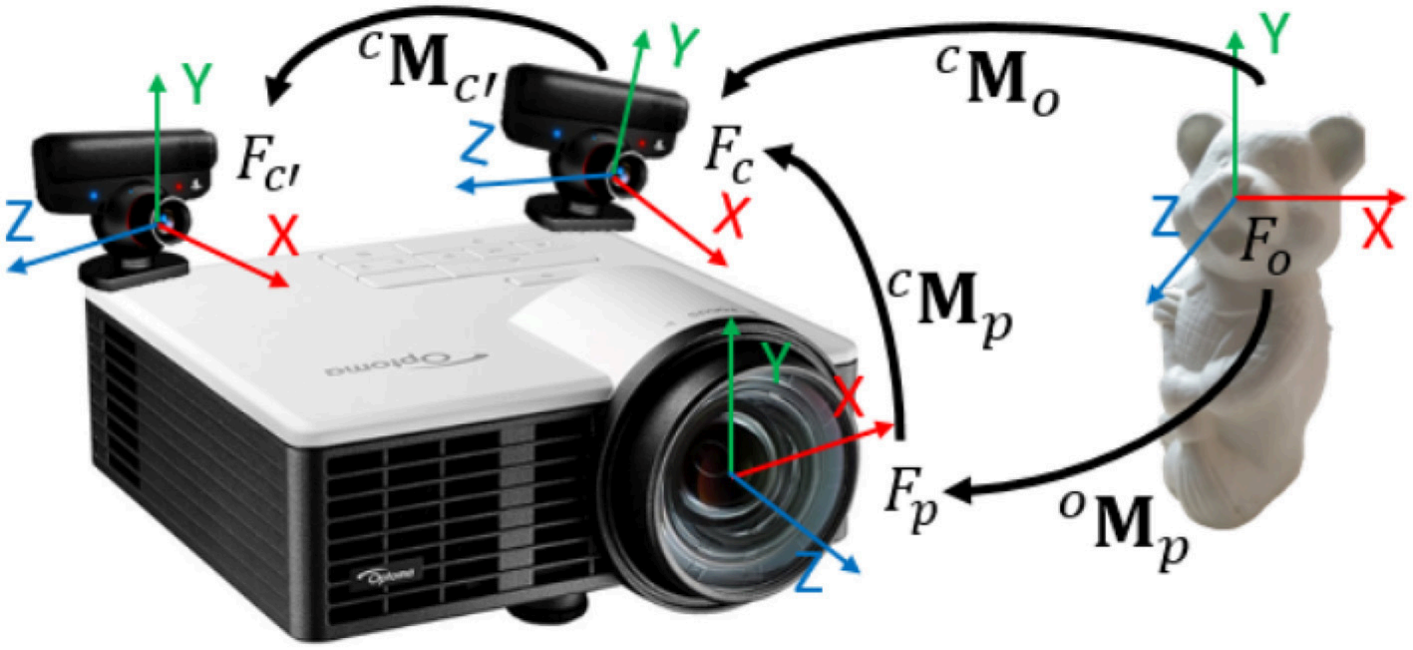
Frame configuration for the calibration process. The estimation of ^*c*^$M_{c^{\prime }}$ is explained in section 4.1, ^*c*^$M_{o}$ is given by the tracking system and ^*o*^$M_{p}$ is computed as explained in section 4.2.2.

Figure 7 illustrates the different frames (F) and transformations (**M**) that take part in the projector pose estimation process.

#### 4.2.3. Virtual scene generation

The virtual scene is generated as a reconstruction of the real scene. This reconstruction is possible thanks to the tracking data and the 3D shape of the objects. The tracking data enables to position the 3D models of the tangible objects in the virtual scene. The projector projection model and pose are applied to the virtual camera in the virtual scene. Thus the relative poses between the 3D models and the virtual camera match as close as possible the real poses between the projector and the tangible objects. The virtual scene is rendered in the virtual camera and the projector projects the rendered image over the real scene. If the different estimation steps of Figure 6 are correctly performed then the projection perfectly matches the real scene.

### 4.3. Interaction tools

**Figure 8:**
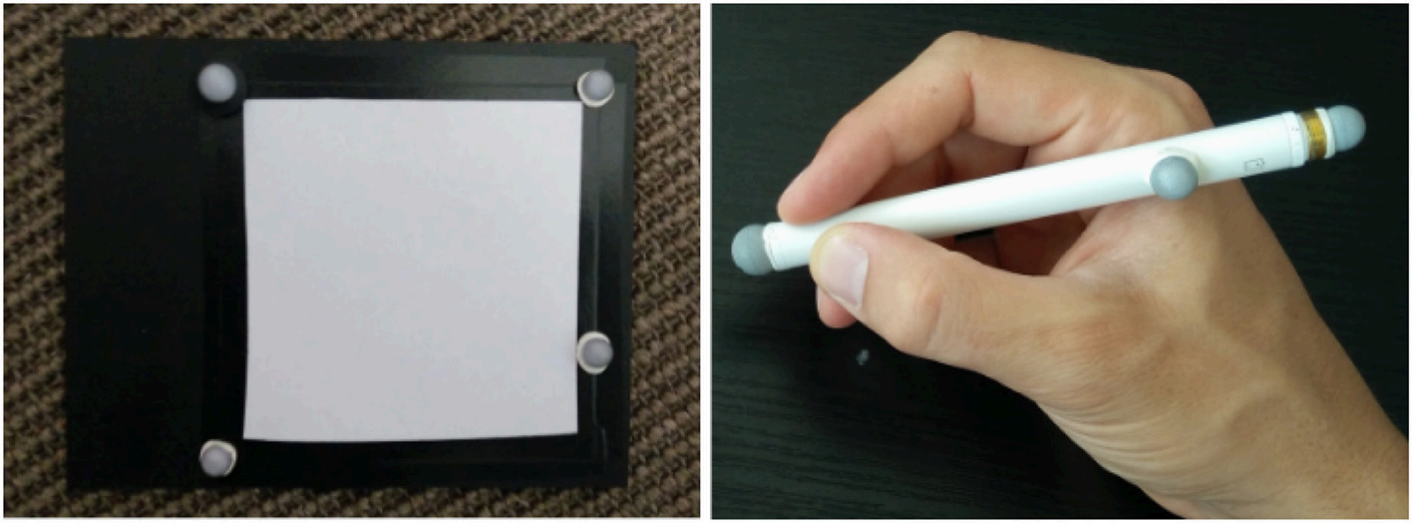
Interaction tools of MoSART: the Panel **(Left)** and the Stylus **(Right)**.

Tangible tools enabling interaction techniques in SAR scenarios have been proposed by Marner et al. (2009) and Marner and Thomas (2010). For our MoSART prototype we have specifically designed two tangible interaction tools. The first tool is the “Panel” (Figure 8, left). It is a squared white board used to display information. The second tool is the “Stylus” (Figure 8, right) which looks like a pen.

**Figure 9:**
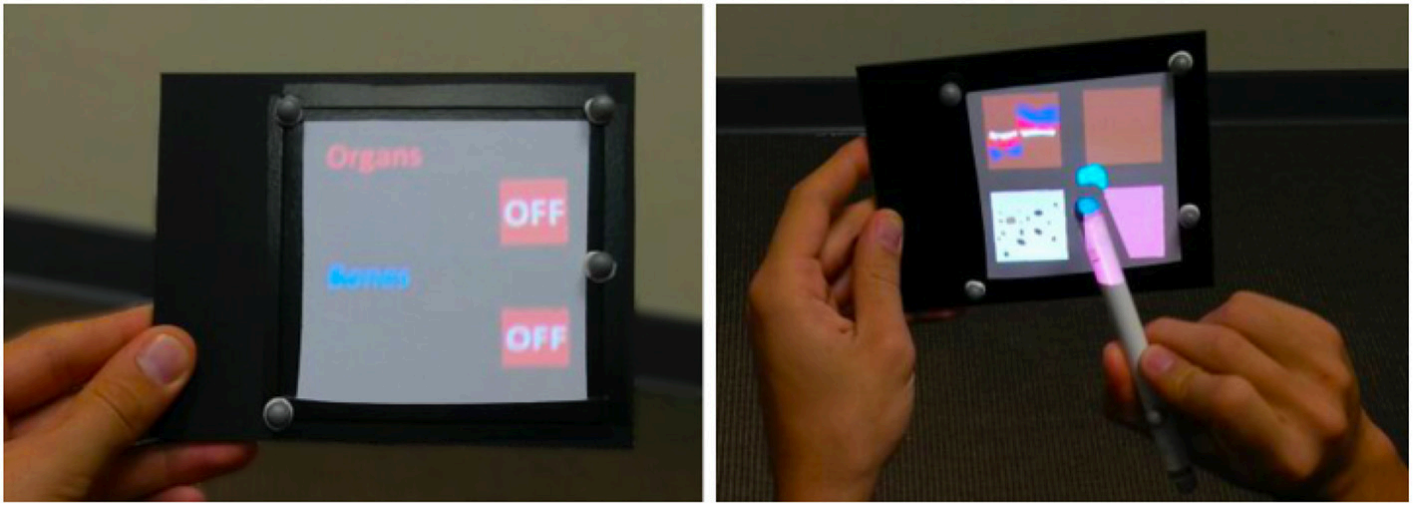
Interaction tools in use: here the Panel **(Left)** is used to display the contextual items of a 2D menu that can be selected by pointing with the Stylus **(Right)**.

Several 3D interaction techniques have been designed to exploit these two tools within MoSART:

1. The **interactive Panel** is primarily used as a control screen (Figure 9, left). It can be straightforwardly used to dynamically display 2D menus with various items. It can also be used as a specific tool, such as: a magnifying glass (Brown et al., 2003), an “x-ray” visualizer, etc.
2. The **interactive Stylus** is primarily used as a 3D pointer. The stylus serves as a selection tool in order activate options and select items by touching them on the control panel (Figure 9, right). But it can also be used as a specific tool as well, such as: a painting tool, a light torch, a laser beam, etc.

The user interactions are taken into account in the virtual scene generation so to modify the content projected over the tangible objects and tools. Other usages of these tools are depicted in the use cases presented later in section 5. Of course, other tangible tools and interaction techniques could be added in MoSART in the future.

### 4.4. Adding collaboration

An main advantage of MoSART is that it can be used in presence of multiple users. Indeed when only one user is equipped with a MoSART headset any external person can still watch the augmented tangible object and exchange orally with the main user. The other persons can also manipulate the tangible object and/or the interaction tools, although being constrained to remain in the workspace of the main user corresponding to the field-of-view of the head-mounted projector. Such collaboration mode could be useful in the context of education/training scenarios in which an external user shares information with the main user.

An extension with multiple devices, which we have not implemented yet, is discussed in section 7. This mode could enable several users to be equipped with MoSART headsets (see a photomontage illustration in Figure 14).

### 4.5. Characteristics and performances

The main characteristics of our MoSART prototype are summarized in Table 1. The system performances have been computed using an MSI GE72 2QE laptop (CPU core I7 2.70GHz, 8Go RAM, SSD, GPU Nvidia GTX965M).

**Table 1:** Main characteristics of the MoSART prototype.

**Characteristic**	**Value**
Weight	1 kg
FOV (H × V)	61° × 38°
Tracking accuracy	0.1 mm
Jitter	±0.08 mm
End-to-end latency	60 ms
Resolution	1,280 × 800
Contrast	20,000:1
Brightness	800 Lumens

The overall weight of the headset is around 1 kg, corresponding to: 472 g for the projector, 170 g for each camera, and around 200 g for the helmet. The prototype currently runs on a laptop PC which can be worn in a backpack. Future work would be needed to further miniaturize the components and embed the computation and the battery directly in the headset.

The projector provides a short-throw ratio of 0.8:1.0 equivalent to an effective field of view (FOV) of 61° × 38° with an image resolution of 1,280 × 800 pixels. The projector provides images with a maximal brightness of 800 Lumens and a contrast of 20,000:1 which ends up with better performances when using the device at an arm distance (between 0.3 and 0.7 m) and acceptable ones when projecting on large objects that are further away (e.g., a car mock-up at a scale close to 1).

The overall latency of the system depends on the tracking and projection display performances. The tracking system runs at 60 Hz and the cameras at 150 Hz ending up with a latency of 10 ms. The end-to-end latency of MoSART, computed as the time between the start of an object motion and the beginning of the corresponding projection motion was found of nearly 60 ms (including rendering and display latencies). The tracking induces a small jitter on the final system performances. Jitter was measured by leaving a tangible object at a stationary position and recording its pose during 600 measurements without filtering process. The object was placed at an arm distance of the cameras (40–50 cm). The mean squared distance of the different computed poses from their mean-normalized center then equals 0.08 mm and the 95% confidence radius of the distribution lies at 0.15 mm.

## 5. Use cases

The MoSART approach offers numerous possibilities in terms of interaction and visualization for single and/or collaborative situations. In this section, we present two different use cases designed and tested with our prototype, for: (1) virtual prototyping and (2) medical visualization purposes. (see accompagnying Video S1).

### 5.1. Virtual prototyping

MoSART enables to augment physical mock-ups with an infinite number of virtual textures. The users become able to interact with the mock-up directly, editing and visualizing the textured variants of the same object.

**Figure 10:**
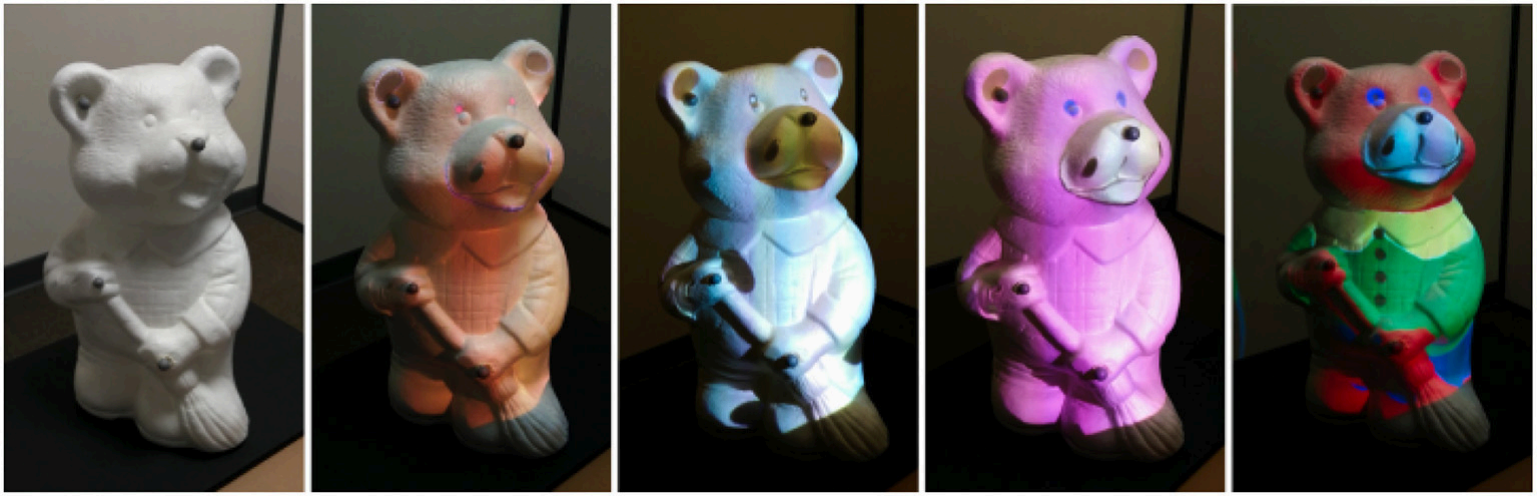
Several textures can be applied to an object for virtual prototyping purpose. The original teddy bear tangible object (left) is augmented with various textures selected with the control Panel.

In our scenario, the user intends to choose the most suitable visuals and dressing of a teddy bear (see Figure 10). This use case could of course be transposed to other kinds of 3D objects, such as for the automotive or clothes industry. The user can switch between different textures that are applied to the tangible object. The selection of textures is made using a 2D menu displayed over the interactive Panel. A previsualization of each available texture is displayed on the Panel (Figure 9, right). The selection is achieved by pointing in the Panel's right location with the interactive Stylus.

**Figure 11:**
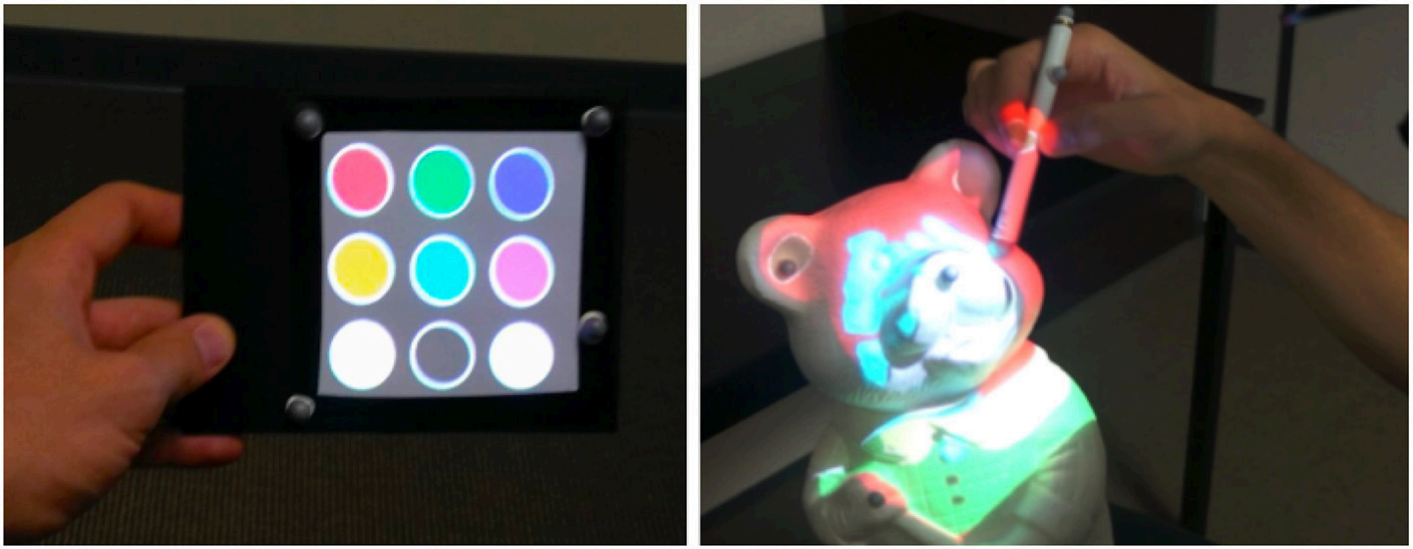
Virtual painting. The user can select a color on the Panel **(Left)** and paint the tangible object with the Stylus **(Right)**.

Second, the user becomes able to edit and change the texture by applying virtual painting over the tangible mock-up. Our interaction tools are also used for this purpose. The interactive Panel is used to display several painting options such as the different available colors (Figure 11, left). The interactive Stylus acts like a paintbrush enabling the user to select a desired color, but also a brush size or a brush shape. Then, the user can directly paint the tangible mock-up with the Stylus as if he/she was painting a statue (see Figure 11, right).

Then, the Figure 1 (right) illustrates how two users can collaborate during the painting task. One user is wearing a MoSART headset and holding the tangible object. The other user is painting the model according to the main user's instructions.

### 5.2. Medical visualization

**Figure 12:**
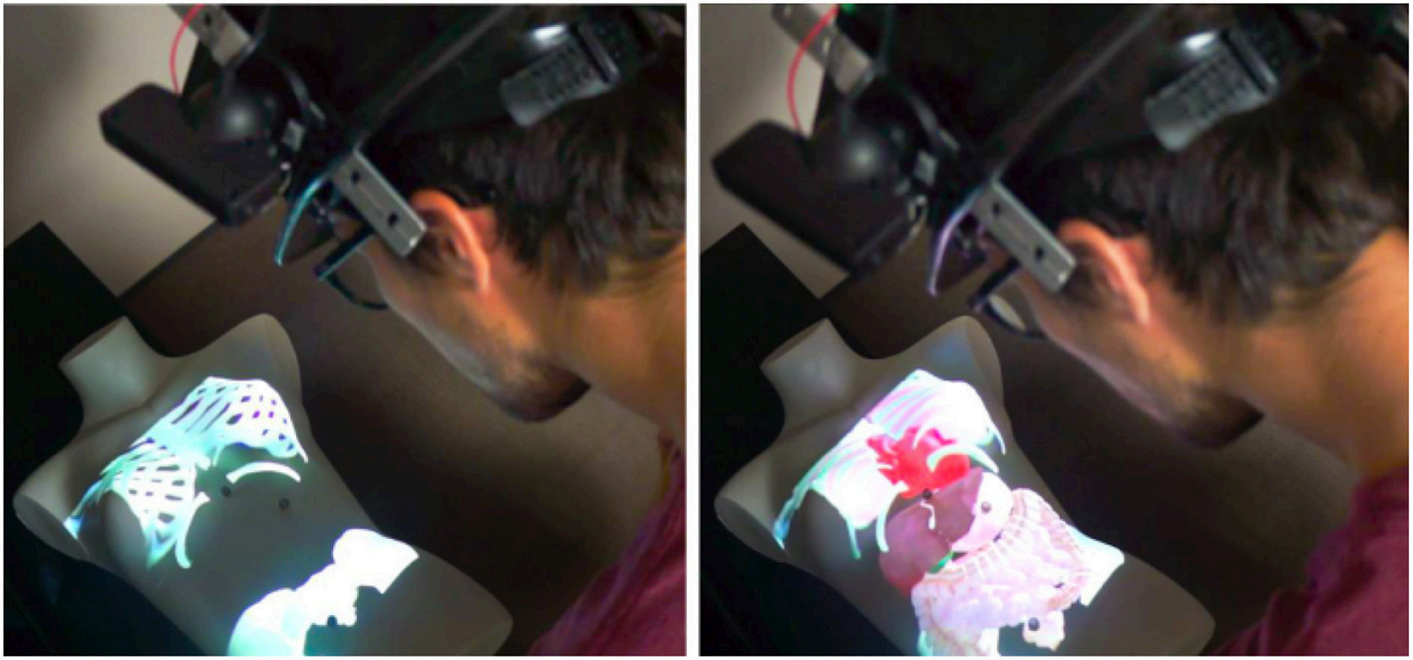
Medical visualization on a tangible chest. The user is able to visualize the bones **(Left)** with or without the organs **(Right)**. [This image is published with the written and informed consent of the depicted individual(s)].

Our second use case is a medical visualization scenario allowing to interact with a tangible body shape. To illustrate this use case, a women chest mannequin is used as a tangible object. The user can visualize different inner components (e.g., bones or organs) positioned with respect to the tangible human body. On Figure 12, the left image illustrates the visualization of the chest bones and the right image illustrates the visualization of both bones and organs of the human chest.

**Figure 13:**
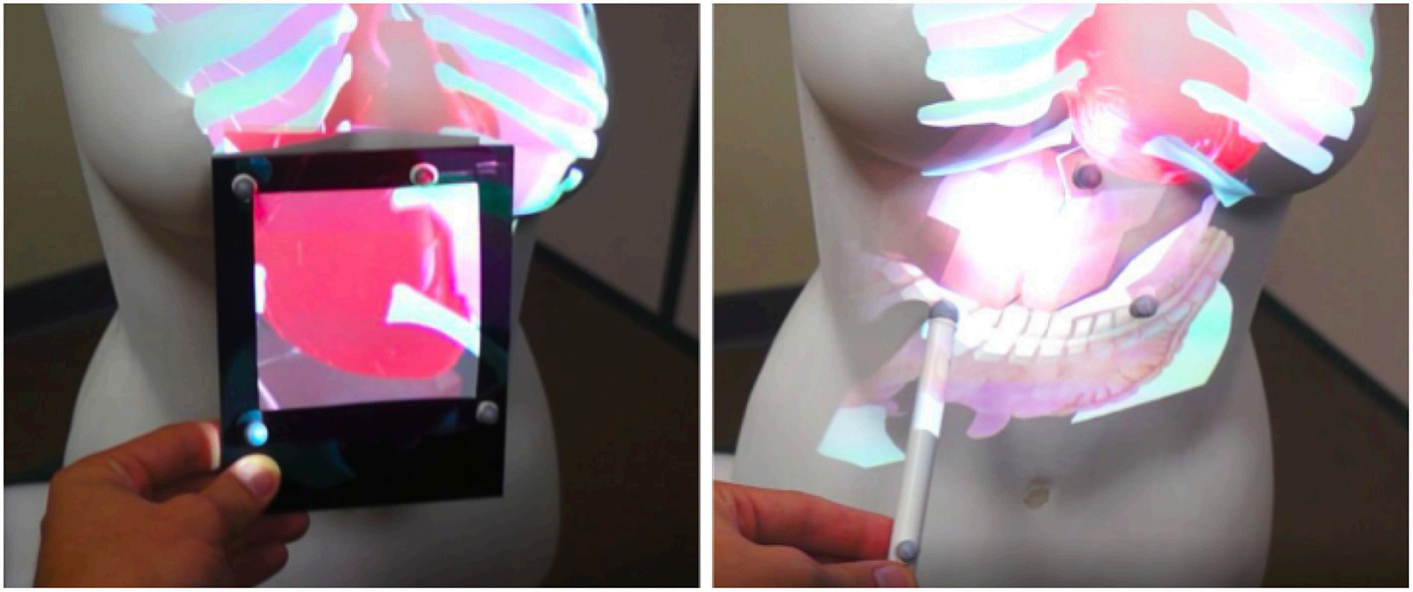
Exploration of 3D medical models. The Panel can be used as a magnifying glass **(Left)** to visualize details or hidden organs. The Stylus can simulate a flashlight **(Right)** to better perceive depths and illuminate some parts of the virtual models.

The interaction tools can first be used to change the visualization state of the application to either: display the bones, the organs, the digestive system or the whole. To do so, the interactive Panel displays a menu with two-state buttons (see Figure 9, left) that the user can toggle with the interactive Stylus used as a pointer. Then, in another interaction mode, the Panel and Stylus can be used to further explore the virtual inner components. The user can notably use the Panel as a magnifying glass (see Figure 13, left) to be positioned in front of an area of interest (such as for observing some small hidden organs of the chest). The Stylus can also serve as a flashlight (see Figure 13, right) to illuminate the organs and have a better perception of their geometry and material.

This visualization use case could inspire similar setups for education or training purposes, in single or collaborative conditions, without being limited to the medical field. Besides, by placing reflective markers over the body of a real person, MoSART could actually be used with a real body and a real patient (Ni et al., 2011). This could be interesting for educational purposes, but also before or during a surgical operation. However, a technical challenge would consist here in accurately tracking the deformable body in real-time.

## 6. Current limitations

There are of course several limitations to our current proof-of-concept of the MoSART approach. We believe that many of them could be solved considering the following path for improvement:

- **Stereoscopic Projection**: In some cases, the MoSART system could benefit from stereo projection. By using a 3D projector and shutter glasses, 3D content could also be projected over the tangible objects. It could provide depth perception such as the one provided by optical see-through AR devices. However, a stereoscopic rendering generally induces the additional need of glasses and might prevent some collaborative scenarios. We can also stress that projecting internal structures on the 3D objects may require to distort the projected image according to the object's model. According to our personal experience the distortion was not very disturbing in our use-cases, although a proper user study would be needed here.
- **Focus Issue**: The focus of the projector can be an issue with our current prototype of MoSART. Indeed since the tangible objects can be manipulated directly, the projection may be done at closer or further distances than the one on focus. This issue can be solved by using either a laser projector or auto-focus algorithms. Nevertheless it could also add some latency to the overall system.
- **Full Portability**: The tracking and projection mapping computations are currently done on an external computer. This computer could be embedded on a backpack together with a battery that could power the projector. The entire system could also be ultimately miniaturized and put inside the headset.
- **Model-based Tracking**: Even though feature-based tracking often provides better performance than model-based tracking, it requires to add markers over the tangible objects and thus preparing the objects beforehand. It could be interesting to test the MoSART approach with a model-based tracking that could provide knowledge about the overall geometry of the real scene and not only of the augmented objects, although maybe to the detriment of the overall performance.
- **Occlusions**: The use of tangible tools can generate partial occlusion problems since the tools can sometimes be located between the projector and the tangible object. The direct manipulation of the objects with the hands can also be a cause of partial occlusions. Partial occlusions generate an incoherent projection over the occluding parts (hands and tools). These issues could be dealt with by detecting occlusions with a depth sensor and then removing the projection over occluding parts. The work from Zhou et al. (2016) proposes already a solution to this problem as long as the occluding objects are not too close to the manipulated object.
- **Resolution**: When projecting over small surfaces (e.g., the interactive panel) the resolution of the image can be rather limited since only a small portion of the projector will be used. Thus displaying detailed information and interacting with small virtual objects over these surfaces can be difficult. A solution to overcome this limitation could be to use a real interactive tablet.

## 7. Discussion

MoSART provides mobile spatial augmented reality on tangible objects. MoSART has been tested within several use cases. Within informal tests it was found very promising in terms of 3D interactions, both for direct manipulation of the physical mock-ups, as well as by means of our dedicated interaction tools.

Considering the current limitation of a majority of AR systems regarding the field-of-view, it is noteworthy that MoSART can considerably increase the FOV and the interaction workspace, especially compared to OST-AR (e.g., Microsoft Hololens). The weight of the first MoSART prototype still remains above the usual weight of commercial OST-AR headsets (less than 600 g for the Hololens for instance). But the design of our prototype has not been fully optimized and miniaturized yet, and we can anticipate a reduction of the total size and weight in the following versions. Future studies could be carried out in order to compare the MoSART approach to other existing head-mounted AR systems (e.g., OST-AR or VST-AR).

**Figure 14:**
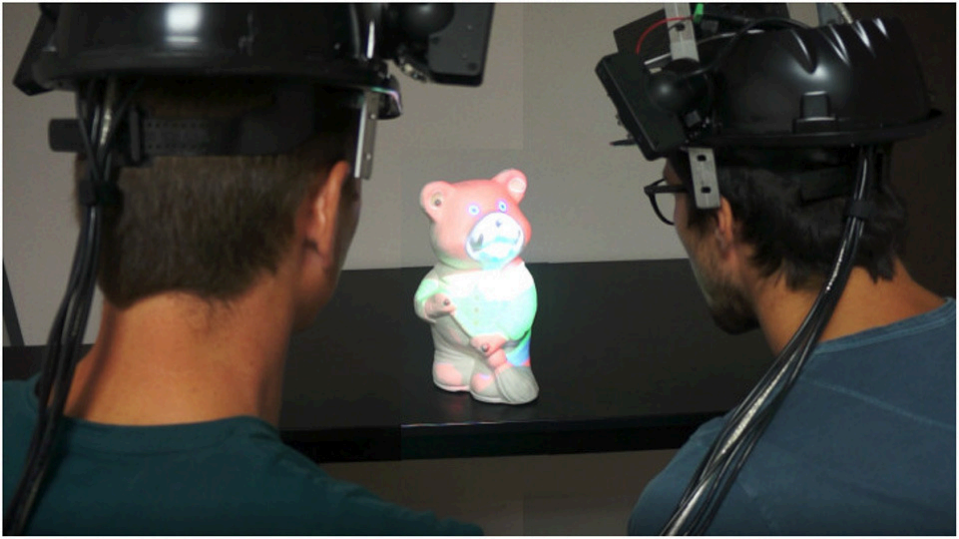
Concept of a collaborative setup with two MoSART systems (photomontage). The users could look at different sides of the object for an even wider projection space. [This image is published with the written and informed consent of the depicted individual(s)].

Regarding the technological evolution of MoSART, we envision several paths for future works in addition to the ones presented in the previous section. The calibration process could first be fully automatized using a similar technique as the one proposed by Moreno and Taubin (2012) which could also improve the 2D-3D matching performance of our system. Then, as mentioned in section 4.4, a multi-user collaboration could be implemented to support several MoSART systems at the same time (see photomontage of Figure 14). In this case a master computer can handle the rendering of the virtual scene in the different virtual cameras corresponding to the different MoSART projectors enabling to generate the virtual scene only once and to avoid inconsistencies when projecting. Such implementation may required to have a blending of the multiple images and the jitter of the distinct devices should still be taken into account. Moreover, if the users are facing each other there is a risk of potential blindness due to the projection light. All these potential issues could thus be investigated in future works.

MoSART has only been tested with rather small tangible objects that can be held in the hand. Thus, it could also be interesting to test the MoSART concept with medium (chairs, tables, etc) and large objects (cars, rooms, etc.). Using MoSART on larger objects might notably require an improvement of the projection performance to keep bright and contrasted projections. A projector could actually be designed specifically to overcome these potential limitations. It could also focus on reducing the overall weight of the system to further facilitate its usage and increase user comfort. Also, a user study could be carried out to evaluate the performances of MoSART in terms of user experience and comfort in comparison with stationary SAR and OST-AR systems.

## 8. Conclusion

We have proposed a novel approach for SAR with tangible objects called MoSART. A proof-of-concept has been designed based on a “all-in-one” headset providing head-mounted projection an feature-based stereo optical tracking. Tangible objects with complex geometries could be augmented with virtual textures, and the user can interact with them thanks to a set of tangible interaction tools. Our prototype shows good performance compare to current AR systems, and it has been tested within several use cases for virtual prototyping and medical visualization. Taken together, our results suggest that the MoSART approach enables a straightforward, mobile, and direct interaction with tangible objects, for a wide range of augmented reality applications in single or collaborative conditions.

## Author contributions

GC, EM, GB, and AL contributed conception and design of the system. GC wrote the first draft of the manuscript. GC, EM, and AL wrote sections of the manuscript. All authors contributed to manuscript revision, read and approved the submitted version.

### Conflict of interest statement

GB is the CEO and Founder of the company Realyz and GC is employed by the company Realyz. The remaining authors declare that the research was conducted in the absence of any commercial or financial relationships that could be construed as a potential conflict of interest.

## Footnotes

^2^Microsoft Hololens - www.microsoft.com/hololens - Accessed: 2017-09-04.

^4^CastAR Augmented Reality Glasses - http://en.wikipedia.org/wiki/CastAR - Accessed: 2017-04-11

## Supplementary material

Video S1 MoSART: Mobile Spatial Augmented Reality for 3D interaction on Tangible objects.

## References

[B1] Akşit K. Kade D. Özcan O. Ürey H. (2014). Head-worn mixed reality projection display application, in Proceedings of the 11th Conference on Advances in Computer Entertainment Technology (Funchal: ACM), 11.

[B2] Aliaga D. G. Yeung Y. H. Law A. Sajadi B. Majumder A. (2012). Fast high-resolution appearance editing using superimposed projections. ACM Trans. Graphics 31:13 10.1145/2159516.2159518

[B3] Arun K. S. Huang T. S. Blostein S. D. (1987). Least-squares fitting of two 3-d point sets. IEEE Trans. Pattern Anal. Mach. Intell. PAMI-9, 698–700. 10.1109/TPAMI.1987.4767965 21869429

[B4] Benko H. Jota R. Wilson A. (2012). Miragetable: freehand interaction on a projected augmented reality tabletop, in Proceedings of the SIGCHI Conference on Human Factors in Computing Systems (Québec City: ACM), 199–208.

[B5] Benko H. Ofek E. Zheng F. Wilson A. D. (2015). Fovear: combining an optically see-through near-eye display with projector-based spatial augmented reality, in Proceedings of the 28th Annual ACM Symposium on User Interface Software & Technology (Charlotte, NC: ACM), 129–135.

[B6] Bimber O. Raskar R. (2005). Spatial Augmented Reality: Merging Real and Virtual Worlds. CRC Press.

[B7] Bolas M. Krum D. M. (2010). Augmented reality applications and user interfaces using head-coupled near-axis personal projectors with novel retroreflective props and surfaces, in Pervasive 2010 Ubiprojection Workshop (Copenhagen).

[B8] Brown L. D. Hua H. Gao C. (2003). A widget framework for augmented interaction in scape, in Proceedings of the 16th Annual ACM Symposium on User Interface Software and Technology (Vancouver, BC: ACM), 1–10.

[B9] Cao X. Forlines C. Balakrishnan R. (2007). Multi-user interaction using handheld projectors, in Proceedings of the 20th Annual ACM Symposium on User Interface Software and Technology (Newport, VA: ACM), 43–52.

[B10] Harrison C. Benko H. Wilson A. D. (2011). Omnitouch: wearable multitouch interaction everywhere, in Proceedings of the 24th Annual ACM Symposium on User Interface Software and Technology (Santa Barbara, CA: ACM), 441–450.

[B11] Hochreiter J. Daher S. Nagendran A. Gonzalez L. Welch G. (2016). Optical touch sensing on nonparametric rear-projection surfaces for interactive physical-virtual experiences. Presence 25, 33–46. 10.1162/PRES_a_00242

[B12] Karitsuka T. Sato K. (2003). A wearable mixed reality with an on-board projector, in Proceedings of the 2nd IEEE/ACM International Symposium on Mixed and Augmented Reality (Tokyo: IEEE Computer Society), 321.

[B13] Marchand E. Uchiyama H. Spindler F. (2016). Pose estimation for augmented reality: a hands-on survey. IEEE Trans. Visual. Comp. Graph. 22, 2633–2651. 10.1109/TVCG.2015.2513408 26731768

[B14] Marner M. R. Thomas B. H. (2010). Tool Virtualization and Spatial Augmented Reality. Ph.D. thesis, Citeseer.

[B15] Marner M. R. Thomas B. H. Sandor C. (2009). Physical-virtual tools for spatial augmented reality user interfaces, in Proceedings of the 8th IEEE International Symposium on Mixed and Augmented Reality (ISMAR) (Orlando, FL: IEEE), 205–206.

[B16] Mohring M. Lessig C. Bimber O. (2004). Video see-through ar on consumer cell-phones, in Proceedings of the 3rd IEEE/ACM International Symposium on Mixed and Augmented Reality (ISMAR) (Arlington, TX: IEEE Computer Society), 252–253.

[B17] Moreno D. Taubin G. (2012). Simple, accurate, and robust projector-camera calibration, in 2012 Second International Conference on 3D Imaging, Modeling, Processing, Visualization and Transmission (3DIMPVT) (Zürich: IEEE), 464–471.

[B18] Newcombe R. A. Izadi S. Hilliges O. Molyneaux D. Kim D. Davison A. J. (2011). Kinectfusion: real-time dense surface mapping and tracking, in Proceedings of the 10th IEEE International Symposium on Mixed and Augmented Reality (ISMAR) (Basel: IEEE), 127–136.

[B19] Ni T. Karlson A. K. Wigdor D. (2011). Anatonme: facilitating doctor-patient communication using a projection-based handheld device, in Proceedings of the SIGCHI Conference on Human Factors in Computing Systems (Vancouver, BC: ACM), 3333–3342.

[B20] Okumura K. Oku H. Ishikawa M. (2012). Lumipen: projection-based mixed reality for dynamic objects, in Proceedings ot the 2012 International Conference on Multimedia and Expo (ICME) (Melbourne, VIC: IEEE), 699–704.

[B21] Olwal A. Lindfors C. Gustafsson J. Kjellberg T. Mattsson L. (2005). Astor: an autostereoscopic optical see-through augmented reality system, in Proceedings of the 4th IEEE/ACM International Symposium on Mixed and Augmented Reality (ISMAR) (Vienna: IEEE), 24–27.

[B22] Pintaric T. Kaufmann H. (2007). Affordable infrared-optical pose-tracking for virtual and augmented reality, in Proceedings of Trends and Issues in Tracking for Virtual Environments Workshop, IEEE VR (Charlotte, NC), 44–51.

[B23] Punpongsanon P. Iwai D. Sato K. (2015). Projection-based visualization of tangential deformation of nonrigid surface by deformation estimation using infrared texture. Virtual Reality 19, 45–56. 10.1007/s10055-014-0256-y

[B24] Raskar R. Van Baar J. Beardsley P. Willwacher T. Rao S. Forlines C. (2006). ilamps: geometrically aware and self-configuring projectors, in ACM SIGGRAPH 2006 Courses (Boston, MA: ACM), 7.

[B25] Schmalstieg D. Hollerer T. (2016). Augmented Reality: Principles and Practice. Addison-Wesley Professional.

[B26] Siegl C. Colaianni M. Thies L. Thies J. Zollhöfer M. Izadi S. (2015). Real-time pixel luminance optimization for dynamic multi-projection mapping. ACM Trans. Graph. 34:237 10.1145/2816795.2818111

[B27] Sueishi T. Oku H. Ishikawa M. (2015). Robust high-speed tracking against illumination changes for dynamic projection mapping, in Proceedings ot the 2015 International Conference on Virtual Reality (VR) (Arles: IEEE), 97–104.

[B28] Van Krevelen D. Poelman R. (2010). A survey of augmented reality technologies, applications and limitations. Int. J. Virtual Reality 9(2):1–20. 10.13140/RG.2.1.1874.7929

[B29] Willis K. D. Poupyrev I. Hudson S. E. Mahler M. (2011). Sidebyside: *ad-hoc* multi-user interaction with handheld projectors, in Proceedings of the 24th Annual ACM Symposium on User Interface Software and Technology (ACM), 431–440.

[B30] Yang L. Normand J.-M. Moreau G. (2016). Practical and precise projector-camera calibration, in Proceedings of the 2016 IEEE International Symposium on Mixed and Augmented Reality (ISMAR) (Merida: IEEE), 63–70.

[B31] Zhou Y. Xiao S. Tang N. Wei Z. Chen X. (2016). Pmomo: projection mapping on movable 3D object, in Proceedings of the 2016 CHI Conference on Human Factors in Computing Systems (San José, CA: ACM), 781–790.

